# Duration of oral contraceptive use relates to cognitive performance and brain activation in current and past users

**DOI:** 10.3389/fendo.2022.885617

**Published:** 2022-09-20

**Authors:** Isabel Asar Noachtar, Esmeralda Hidalgo-Lopez, Belinda Pletzer

**Affiliations:** Department of Psychology and Centre for Cognitive Neuroscience, University of Salzburg, Salzburg, Austria

**Keywords:** oral contraceptives (OC), navigation, verbal fluency, brain activation and connectivity, androgenicity, duration of OC use, sex hormones, progestins

## Abstract

Previous studies indicate effects of oral contraceptive (OC) use on spatial and verbal cognition. However, a better understanding of the OC effects is still needed, including the differential effects of androgenic or anti-androgenic OC use and whether the possible impact persists beyond the OC use. We aim to investigate the associations of OC use duration with spatial and verbal cognition, differentiating between androgenic and anti-androgenic OC. Using functional magnetic resonance imaging (MRI), we scanned a group of 94 past and current OC-users in a single session. We grouped current OC users (N=53) and past OC users with a natural cycle (N=41) into androgenic and anti-androgenic user. Effects of OC use duration were observed for current use and after discontinuation. Duration of OC use was reflected only in verbal fluency performance but not navigation: The longer the current OC use, the less words were produced in the verbal fluency task. During navigation, deactivation in the caudate and postcentral gyrus was duration-dependent in current androgenic OC users. Only during the verbal fluency task, duration of previous OC use affects several brain parameters, including activation of the left putamen and connectivity between right-hemispheric language areas (i.e., right inferior frontal gyrus and right angular gyrus). The results regarding performance and brain activation point towards stronger organizational effects of OCs on verbal rather than spatial processing. Irrespective of the task, a duration-dependent connectivity between the hippocampus and various occipital areas was observed. This could suggest a shift in strategy or processing style with long-term contraceptive use during navigation/verbal fluency. The current findings suggest a key role of the progestogenic component of OCs in both tasks. The influence of OC use on verbal fluency remains even after discontinuation which further points out the importance of future studies on OC effects and their reversibility.

## Introduction

It is well documented that endogenous ovarian hormones affect women’s brain structure, function and connectivity, resulting in either the maintenance or alteration of various cognitive and emotional functions in fluctuating hormonal milieus (1–3). In particular, the hippocampus and amygdala, as well as the basal ganglia and pre-frontal cortex appear to be particularly sensitive to the effects of endogenous ovarian hormones. For example, it has been repeatedly demonstrated, that the volume (4–6), activation (7) and connectivity (5, 7) of the hippocampus are increased during phases of high estrogen. Vice versa, fronto-striatal networks appear to be sensitive to progesterone, since increased basal ganglia volumes (6; Pletzer et al., 2018), fronto-striatal activation (7) and alterations in connectivity of caudate, putamen and dorsolateral prefrontal cortex (DLPFC) (7–10) have been repeatedly demonstrated. While the effects on the hippocampus and frontal cortex are plausible due to their high density in sex hormone receptors (11–15), the effects by which sex hormones affect the basal ganglia are less understood. However, it has been well documented that sex hormones affect a variety of neurotransmitter systems, including dopaminergic transmission (16).

Combined oral contraceptives (OCs) contain synthetic analogs of endogenous ovarian hormones, including a synthetic estrogen, mostly ethinyl-estradiol, and one of various synthetic progestins. These hormones exert their contraceptive actions *via* activation of the estrogen and progesterone receptors. Accordingly, it is plausible that OCs also influence brain structure, function and connectivity (17–20). Nevertheless, our knowledge about the effects of OCs on the female brain and behavior is still limited. The majority of studies have compared OC users to non-users (20) and a very small number of prospective studies have reported short-term effects of contraceptive use on the brain (21–24). Likewise, potential long-term effects are not well understood and it is still unknown if possible neurocognitive effects are reversible. While the effects of estradiol and progesterone along the female menstrual cycle are short-lived, OC intake usually spans 21-24 days, interrupted by a 4-7 day withdrawal period. Furthermore, synthetic steroids have a higher potency and longer half-life than endogenous hormones (25). It is thus possible that effects accumulate over different treatment cycles. On the other hand, endogenous steroid hormones are suppressed by oral contraceptives (26). Additionally, increased sex hormone-binding globulin levels were found in current OC users compared to never-users (27). In this scenario, it is still unclear which mechanism might be responsible for cognitive changes. We expect our results to be due to a combination of the endogenous suppression and the synthetic hormone intake by OC use.

This is especially of interest since millions of women worldwide take oral contraceptives for extended time periods, spanning years if not decades during their reproductive period. So far, the effects of OCs on brain and behavior are inconsistent and fragmentary. Some studies found an association between OC use and impaired cognitive performance (28), other studies found an improve in performance (29, 30) and some do not find any relationship between OC intake and cognitive performance (31, 32). Furthermore, the reversibility of potential cognitive effects is still unknown. This lack of knowledge raises concerns and questions regarding possible cognitive effects which are unintended side effects of the pill. The lacking evidence of possible OC effects may cause great uncertainty for women starting or using OCs (33). It is, therefore, important to gain a better understanding of the OC effects on the female brain.

One approach to study the long-term effects of OC treatment retrospectively is to focus on the effects of OC treatment duration. While studies using this approach are correlational and cannot make causal inferences, they may generate hypotheses for more costly and time-consuming prospective studies. So far, only a few studies have investigated the effects of OC use duration on brain and cognition (34–36). One study on past users of hormonal contraceptives found an association between longer pill intake and better performance during a speed and flexibility task (34). The authors also observed a tendency of better visuospatial abilities with longer intake duration (34). Another study investigating the impact of pill duration in naturally cycling women found duration of previous OC use positively correlated to gray matter volumes of the hippocampus and basal ganglia (7). When controlling for the time since OC discontinuation, the hippocampal volume effects disappeared but not the effects on the basal ganglia. Both studies question the immediate reversibility of OC effects on brain structure and behavior, but hint at potential neuroprotective effects of OC even after discontinuation of OC use. In current OC users, gray matter volume of bilateral fusiform gyri, hippocampus, parahippocampus, middle frontal gyri and anterior cingulate cortex was found to relate positively to pill duration (35). This effect was dependent on another property of synthetic progestins, their androgenicity, i.e., their actions at the androgen receptor.

Oral contraceptives of older generations exert androgenic actions because they are derived from 19-nortestosterone and, therefore, have a higher binding affinity to androgen receptors compared to newer progestins derived from spironolactone or progesterone (37). These newer progestins (e.g., chlormadinone acetate) bind specifically to progesterone receptors which lead to anti-androgenic actions (37). Unfortunately, only few studies of OC actions on the brain have accounted for the different properties of different progestins. However, given that androgens are responsible for a variety of organizational and activational effects on brain structure and function, agonistic vs. antagonistic actions of progestins at the androgen receptor may result in opposing effects.

Spatial and to a lesser extent also verbal functions have been particularly extensively studied with regards to testosterone actions, since the robust sex differences in these functions make them a likely candidate for organizational sex hormone actions (38, 39). Interestingly, neuroimaging studies focusing on the differential processing patterns underlying these sex differences, found the inferior frontal gyrus (IFG) differentially activated in men and women during both, verbal and spatial processing (40, 41). Results regarding the activational effects of testosterone during adulthood are more controversial (42), but point to an improvement of spatial and decline of verbal functions with higher circulating testosterone levels (though an inverted U-shaped relationship has also been discussed). For example, several studies demonstrated better navigation performance in participants with higher circulating testosterone levels (Burkitt et al., 2007; Müller et al., 2016; but see Nowak et al., 2014). Vice versa, higher levels of testosterone were associated with reduced word production during a verbal fluency task (43) and verbal recall (44) in women (but see 45, 46). Most convincing evidence comes from studies using testosterone administration. Administration of one dose of 0.5 mg sublingual testosterone already improved performance during a mental rotation task compared to a placebo (47) and increased medial temporal lobe activation during successful virtual navigation (47). Conversely, half a year of testosterone treatment in postmenopausal women led to decreased left IFG activation during a verbal fluency task, though no change in task performance (48). Accordingly, androgen administration appears to alter verbal and spatial processing in women. It is thus plausible that progestins with androgenic vs. anti-androgenic properties also have opposing effects on verbal and spatial processing.

Indeed, there is some indication from cross-sectional studies that the use of androgenic OCs is associated with improved spatial abilities (49). Subdividing OC-users in androgenic, less androgenic and anti-androgenic groups revealed improved mental rotation performance in androgenic OC-users compared to anti-androgenic OC users, as well as non-users (49). Another study supported this finding by demonstrating that women taking androgenic OC had an advantage in a mental rotation task compared to non-users (18). In contrast, anti-androgenic OCs seem to be associated with improved verbal fluency performance. Users of anti-androgenic OCs generated significantly more words in a verbal fluency task compared to users of androgenic OCs (50). However, to the best of our knowledge no study has compared the duration effects of androgenic and anti-androgenic OC use on the neural correlates of verbal and spatial processing.

The present study aims to elucidate the effects of OC use duration on spatial and verbal cognition, while controlling for the androgenicity of the progestin component. Estrogenic and progestagenic effects should result in comparable effects across both groups, effects attributable to androgenic vs. anti-androgenic actions should result in opposite effects across groups. However, cross-sectional comparisons present a methodological problem regarding confounding effects. The tolerance for a specific oral contraceptive or the tendencies to side effects leads to the so-called “survivor effect” (51) and hinder cross-sectional comparison interpretations. We, thus, opted for studying those time-dependent associations that may accumulate over OC use duration, while controlling for the androgenicity of OCs. Furthermore, to disentangle immediate effects from effects that persist after discontinuation of OC use, we included both, current OC-users and naturally cycling women with previous OC-use in our study. For the same reasons aforementioned, group comparisons were avoided as well between these two groups since current users differ from previous users with a natural cycle regarding the hormonal status as well as other factors (e.g. relationship status, sexual activity, personality features, socioeconomic status).

To approach the cognitive effects of duration of OC use, we chose a navigation task and a verbal fluency task for which previous neuroimaging data and results regarding sex differences as well as the effects of endogenous sex hormones are available (7, 40) and focused our neuroimaging analyses on brain areas sensitive to estrogenic, progestagenic and/or androgenic actions as described above, i.e. the hippocampus, caudate, DLPFC and IFG. Based on previous results, we hypothesize that longer androgenic pill use is associated with better navigation performance, whereas longer anti-androgenic pill use is linked to better verbal fluency performance. We expect higher activation in hippocampus, caudate, and DLPFC with longer duration of OC use in both tasks irrespective of group and differential effects of androgenic vs. anti-androgenic treatment duration on the IFG.

## Methods

### Participants

Ninety-four healthy women (53 with current OC use, 41 with past OC use and currently natural cycle) were scanned once during either their menses (past users) or their second or third week of oral contraceptive intake (current users). The participants were subdivided into naturally cycling women with previous androgenic pill use (N=21), previous anti-androgenic pill use (N= 20) and OC users with current androgenic pill intake (N=30) and current anti-androgenic pill intake (N=23). Past OC users had a mean age of 24.61 (SD=3.64) and a mean IQ of 111.17 (SD=7.58) and did not differ significantly in age and IQ between the subgroups of previous androgenic and anti-androgenic users. Current OC users had a mean age of 21.42 (SD=3.28) and a mean IQ of 107.08 (SD=10.62) and differed significantly in age (*t*_(51)_ = -4.10, *p* < 0.0001), IQ (*t*_(46)_ = -2.45, *p* < 0.05) and pill duration (*t*_(51)_ = -2.86, *p* < 0.01) between androgenic and anti-androgenic pill users (**Table 1**). In order to control for the age and IQ of the participants, these variables were used as regressors of no interest (or covariables) in subsequent analyses. Past users discontinued OC intake for at least 6 months and provided information about the last three menses start dates to make sure their last natural cycles were regular. We checked if participants complied with the criteria of Fehring etal. (52), i.e., 21 – 35 days of cycle duration with a maximal deviation of 7 days between the cycle lengths. OC users took the pill at least for 6 months.

**Table 1:** Demographics between past/current and androgenic/anti-androgenic OC users.

	N	Age (years)		IQ		Education	OC use duration (years)	
		Mean	SD	Mean	SD	N	Mean	SD
Current androgenic pill user	30	20.00*	1.44	104.07*	9.86	28 upper secondary, 2 higher education	3.25	1.82
Current anti-androgenic pill user	23	23.26	4.05	111.00	10.48	16 upper secondary, 8 higher education	5.32**	3.39
Previous androgenic pill user	21	24.24	3.32	111.43	7.22	12 upper secondary, 9 higher education	3.97	3.01
Previous anti-androgenic pill user	20	25.00	4.00	110.90	8.12	11 upper secondary, 9 higher education	3.80	3.50

Number of participants (N), age, intelligence quotient (IQ) and duration of OC use in years separated for the four experimental subgroups. *Age and IQ of the current androgenic users was significantly different to the other subgroups. **Duration of oral contraceptive (OC) use in current anti-androgenic users differed significantly compared to the other three groups.

We categorized past and current OC users into androgenic pill use and anti-androgenic pill intake depending on the OC’s progestin. We classified all progestins derived from 19-nortestosterone (except for dienogest) as androgenic because either they or their metabolites (most are rapidly metabolized to Levonorgestrel) demonstrate a binding affinity to the androgen receptor. Progestins derived from spirobolactone or hydroxy-/19-norprogesterone as well as dienogest are classified as anti-androgenic pills. Dienogest is derived from 19-nortestosterone but it exhibits highly selective binding to the progesterone receptor and exerts anti-androgenic activities (53). OC users with levonorgestrel, desogestrel, gestodene, etonogestrel, norelgestromin were counted among androgenic progestins; and dienogest, drospirenone, chlormadinone acetate, cyproterone acetate, nomegestrol acetate as anti-androgenic progestins. Participants had no history of psychological, endocrinological or neurological illness and showed no brain tissue abnormalities. Given that adolescence is a developmental phase, in which the brain seems to be especially sensitive to effects of synthetic steroid hormones (54), we explored the effects of age of first intake in our study. Except for two participants, our sample initiated OC use during adolescence. Since results did not change when those two participants were excluded, we kept the two subjects in our sample.

### Ethics statement

The study was approved by the University of Salzburg’s ethics committee. Subjects gave their informed written consent to participate in this study. The methods conformed with the Declaration of Helsinki.

### Procedure

Before the magnetic resonance imaging (MRI) appointment, a pretest was conducted during which the participants signed the informed consent and completed a training of the cognitive tasks. Additionally, they filled in a screening questionnaire and the Advanced Progressive Matrices by Raven (55). The task-based fMRI was included in a larger session that started with a functional resting-state sequence, followed by task-based scans, high-resolution structural scans and diffusion weighted scans. The MRI session took approximately 1 hour and 30 minutes. Testing sessions were scheduled during the first to seventh day of menses in past users and during the second or third intake week in current OC users. OC duration was based on self-reports and endogenous hormone levels were not analyzed. Oral contraceptive users show reduced level of ovarian hormones which do not reflect the activity of the exogenous hormones at the receptors. Thus, those values would not be informative and were not analyzed in the present work.

### Navigation task

The navigation task consisted of 10 levels, for which participants had to navigate through a 3D virtual environment and reach as many goals as possible within 30 seconds. The task was adapted from an earlier version by reducing it from 20 levels to 10 levels (56). The 3D-environment was designed a 10 x 10 field matrix with one of ten different landmarks located on each field. Specific landmarks consisted of a tree, flowers, bushes, bench, stone, house, church, stairs, traffic light and a bridge. In each row and column all ten different landmarks were placed once.

Each level began on a starting field outside of the environment. After a countdown the current cardinal direction they were facing was presented as well as the directions to the first goal. These instructions always contained information about the cardinal direction as well as the landmark of the goal field. As soon as the subject reached the first goal, the directions to the second target location were presented. We presented the levels of the navigation and verbal fluency task alternately: the cognitive tasks started with a navigation level and continued with a verbal fluency level.

### Verbal fluency task

The verbal fluency task consisted of 10 levels in which participants had 30 seconds per level to think of as many words as possible belonging to a presented semantic category (e.g. farm animals). The consecutive words, which they mentally listed, should relate to the same subcategory. When they could not think of more words within a subcategory, they should continue with words of the next subcategory. To measure the behavioral performance, subjects had to click a remote-control button for each word within a subcategory. As soon as they continued with words of a new subcategory, they had to switch the button. We asked to silently generate the words (covert verbal fluency task) to avoid movement artefacts. It should be noted that neuronal activity during covert and overt verbal fluency might differ (57, 58). Therefore, our results should be interpreted within a framework of covert word production. During the pretest training participants had to vocalize the words that came to their mind to check if they did the task correctly. The presented categories were prescreened for overall difficulty (number of words produced if no specific instruction is given) and clustering difficulty (number of words produced under the clustering instruction) in a sample of 45 men and 45 women (see 41).

### fMRI acquisition

The fMRI data were acquired on a Siemens Prisma fit 3.0 Tesla scanner at the Christian Doppler clinic in Salzburg, Austria. A T2-weighted gradient echo planar (EPI) sequence sensitive to BOLD contrast was used for the task-based functional scan (TR = 2250 ms, TE = 30 ms, FOV 192 mm, matrix size 192 × 192, slice thickness = 3.0 mm, flip angle 70°, voxel size 3.0 × 3.0 × 3.0 mm, 36 transversal slices parallel to the AC-PC line). To acquire the high-resolution structural images a T1-weighted sagittal 3D MPRAGE sequence was used (TR = 2300 ms, TE = 2.91 ms, TI delay of 900 ms, FOV 256 mm, slice thickness = 1.00 mm, flip angle 9°, voxel size 1.0 × 1.0 × 1.0 mm, 176 sagittal slices).

### fMRI data analysis

The first 6 images of each scanning session were discarded during the preprocessing and the remaining scans were despiked using 3d-despiking as implemented in AFNI (afni.nimh.nih.gov). We used the Statistical Parametric Mapping software (SPM12) to realign and unwrap the images and extract six movement parameters. A biophysically-based model (Functional Image Artefact Correction Heuristic, FIACH) (59) was applied to identify and correct for non-physiological noise. We filtered the images and extracted six regressors of physiological noise *via* principal components analyses from a time-series signal-to-noise ratio (TSNR) map. Furthermore, the standard SPM12 procedures for preprocessing were used including the slice-timing, co-registration of functional to structural images, segmentation of structural images using the computational anatomy toolbox for SPM (CAT12) and normalization of functional images using the parameters obtained by CAT12. Afterwards, the data was resampled to isotropic 3 × 3 × 3 mm voxels and smoothed with a Gaussian kernel of 6 mm.

For the subject-dependent fixed-effects first-level analysis, two regressors of interest were modelled separately to predict blood-oxygen-level-dependent-imaging (BOLD) responses to the different types of events: navigation and verbal fluency with clustering instruction. The following regressors of no interest were entered to the models: episodes during which instructions appeared on the screen, the six realignment parameters and the six physiological noise parameters obtained from the FIACH procedure. All regressors were obtained by convolving the duration of the event with the canonical hemodynamic response function implemented in SPM. A high pass filter cut-off was set at 128s and autocorrelation correction was performed using an autoregressive AR(1) model (60).

For the first level analysis, one statistical contrast was defined for each of the two regressors of interest to compare BOLD-response during the tasks to baseline (blank screen). This resulted in two contrast images (activation maps), one for navigation and one for verbal fluency for each subject. Further, the contrasts were scaled (61) by dividing the contrast image by the amplitude of low-frequency fluctuation (ALFF) map (62) using the Data Processing for Analysis of Brain Imaging (DPABI) toolbox (63) as well as a band-pass filter of 0.01–0.08Hz.

At the second level, we first focus on a region-of-interest-based (ROI) analysis and then explore the whole brain level. For the ROI analysis, we entered the first-level scaled contrast images from each subject and task into two one-sample t-test, separated for navigation and verbal fluency. We extracted the eigenvalues as measures of BOLD- response from the following bilateral regions of interest (ROIs) for both tasks: hippocampus, caudate, IFG (BA 44/45) and DLPFC (BA46). The ROIs were defined using masks based on Brodmann-areas (BA), which were implemented in the Wake Forest University (WFU) Pickatlas toolbox (64). Subsequent linear models with these variables as dependent variables were run as detailed in section statistical anaylsis.

Additionally, we explored whether duration of OC use affected brain activation at the whole brain level. Therefore, we entered the contrast images into four different full factorial designs; separated for past and current OC users as well as navigation and verbal fluency. For each model, duration of OC use, age and IQ were introduced as regressors and we modelled their interaction with group (androgenic vs. anti-androgenic). For the second level, results were masked with a SPM gray matter template and uncorrected primary threshold of p < 0.001 and secondary cluster-level FWE-corrected threshold of p < 0.05 were used.

### Connectivity analysis

In order to investigate the connectivity of each ROI, bilateral hippocampi, caudate, IFG and DLPFC were used as seeds for the seed-to-voxel connectivity analysis in CONN-toolbox (65). Linear detrending for white matter (WM) and cerebrospinal fluid (CSF) influences, a band-pass filter (0.008-0.09 Hz) and additional motion-correction were executed on the preprocessed functional images. The voxel-wise connectivity maps of each participant for each ROI (hippocampus, caudate, DLPFC, IFG) were entered into a full factorial design separated for past and current users as well as navigation and verbal fluency. For each model, duration of OC use, age and IQ were introduced as regressors of no interest and we modelled their interaction with group (androgenic vs. anti-androgenic). In order to account for multiple testing, the uncorrected p-value threshold was divided by the number of ROIs and therefore set to p < 0.000125. Results were masked with a SPM gray matter template, and reported when Family-Wise Error (FWE) corrected p < 0.05. For the ROI-to-ROI connectivity analysis, Z-scores were extracted in order to analyze the interhemispheric connectivity of hippocampus, caudate, DLPFC and IFG.

### Statistical analysis

We used R Version 1.4.1717 and SPSS Version 26 to compute the statistical analysis. Linear mixed models (LMMs) were performed by using the lm or lmer function from lme4 package (66) in order to assess the duration of OC use*androgenicity interactive effect on the following dependent variables: i) performance (words produced for verbal fluency, goals reached for navigation), ii) BOLD response, iii) interhemispheric ROI-to-ROI connectivity. Apart from *duration of OC use* and *androgenicity*, which were the factors of interest, in every model, we included age and IQ in order to control for these variables (e.g., performance ~ duration of OC use*androgenicity + *age* + *IQ*). For the models including ROI’s brain activation, we additionally controlled for hemisphere (e.g., navigation task IFG ~ duration of OC use*androgenicity + *hemisphere* + *age* + *IQ*). In case no significant interaction between androgenicity and pill duration was observed, the interaction was removed from the model and the main effect of duration of OC use was assessed (e.g., navigation task IFG ~ *duration of OC use* + *androgenicity* + *hemisphere* + *age* + *IQ*). The main effect of androgenicity was not reported as cross-sectional comparisons were not the goal of this study. For the models including brain activation, and ROI-to-ROI connectivity, we accounted for multiple testing further FDR-correcting the p-values for the four bilateral ROIs. All continuous variables were scaled prior to analyses to allow for interpretation of effect sizes based on standard deviations.

## Results

### Behavioral performance

**Figure 1:**
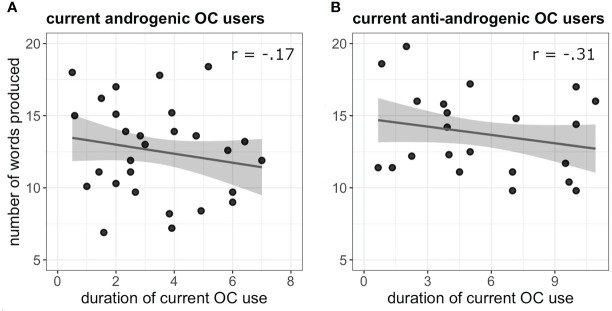
**(A)** Negative association between verbal fluency performance and duration of OC use (in years) in current androgenic users. **(B)** Negative association between verbal fluency performance and duration of OC use (in years) in current anti-androgenic users.

Irrespective of androgenicity, the duration of OC use was not significantly related to navigation performance in current OC users (*b* = -0.04, SE_b_ = 0.17, *t*_(47)_ = -0.27, *p* = 0.79) or past OC users (*b* = 0.14, SE_b_ = 0.22, *t*_(36)_ = 0.64, *p* = 0.52). Irrespective of androgenicity, duration of OC use was negatively related to verbal fluency performance in current OC users (*b* = -0.45, SE_b_ = 0.17, t_(48)_ = -2.60, *p* = 0.013) (**Figure 1**). The longer the current OC use, the less words were produced in the verbal fluency task. In naturally cycling women previous pill duration did not affect the VF performance (*b* = 0.19, SE_b_ = 0.22, *t*_(36)_ = 0.91, *p* = 0.37).

### Brain activation: Overall task-related activation

During the navigation task, and in line with task-related literature (40) we observed significant activation in the bilateral superior frontal gyrus and anterior insula, left superior parietal lobe and supplementary motor cortex (**Table 1**). Specifically, our results show similar activation compared to the activation of the female sub-group in previous samples (40). During verbal fluency, the activation network was left-lateralized showing a large cluster including the bilateral middle cingulate and superior frontal gyrus with a peak in the supplementary motor cortex and supramarginal gyrus (**Supplementary Table 1**). The activation network is in line with previous fMRI-studies on covert verbal fluency (67, 68).

### Brain activation: ROI-analysis

During navigation, activation in the hippocampus, DLPFC or IFG was not significantly related to the duration of OC use, irrespective of the androgenicity, in current OC users (all b < 0.58, all t < 2.58, all p > 0.05).

**Figure 2:**
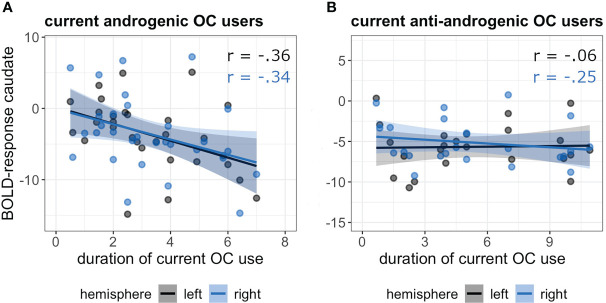
Association between duration of current OC use [in years] and BOLD-response in the bilateral caudate in androgenic **(A)** and anti-androgenic **(B)** OC-users during navigation.

However, there was a significant interaction between the *duration of current OC use* and *androgenicity* on caudate activation (*b* = 0.83, SE_b_ = 0.32, *t*_(53)_ = 2.58, *p*_uncorr. =_ 0.01, *p*_FDR_ = 0.04). While androgenic OC-users showed a significant inverse relation of caudate activation and pill duration, showing a stronger deactivation with longer duration of pill use (r_(55)_ = -0.35, *p* = 0.009); anti-androgenic users showed no pill duration effect (r_(41)_ = -0.15, *p* = 0.33) (**Figure 2**).

During verbal fluency, there was no significant association of pill duration, irrespective of the androgenicity, to activation in any of the ROIs in current OC-users (all b < 0.46, all t < 1.36, all p > 0.05).

There was no significant association between duration of previous OC use, irrespective of the androgenicity, and activation in any of the ROIs during navigation or verbal fluency (all b < 0.45, all t < 1.49, all p > 0.05).

### Brain activation: Whole brain analysis

**Figure 3:**
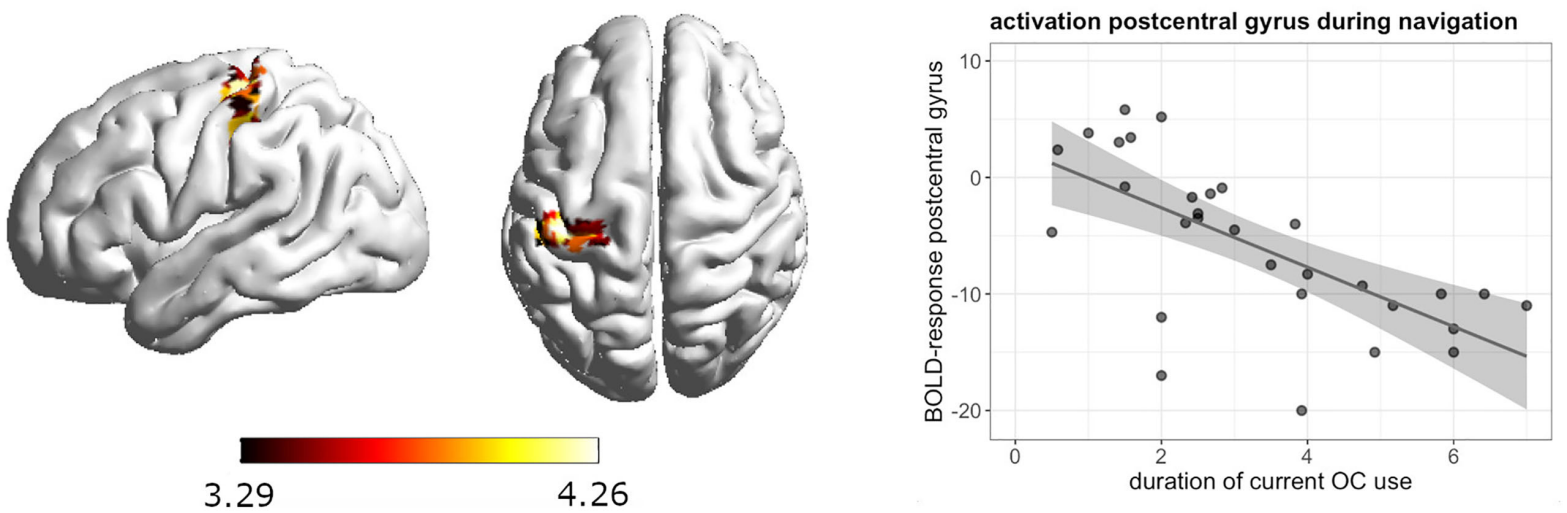
Negative association between duration of OC use and left postcentral gyrus activation during navigation in current androgenic OC users.

**Figure 4:**
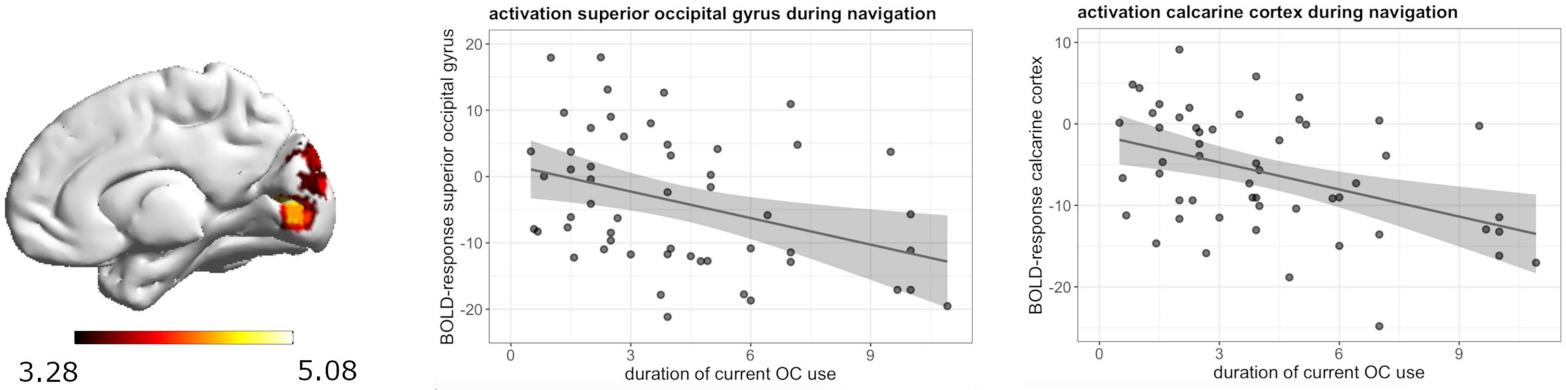
Negative association between duration of OC use and right superior occipital gyrus and right calcarine cortex deactivation during navigation in current OC users.

During navigation, whole brain analyses revealed a significant negative association between current androgenic OC use and activation in the left postcentral gyrus ([-39, -28, 58], 88 voxels, T = 4.26, *p*_FWE_ = 0.008) (**Figure 3**). The longer the duration of androgenic OC use, the stronger was the deactivation in the post-central gyrus. We observed a negative association in androgenic and anti-androgenic OC users between duration of OC use and right superior occipital gyrus activation ([24, -79, 16], 135 voxels, T = 5.08, *p*_FWE_ = 0.001) as well as right calcarine cortex activation ([18, -76, 7], 73 voxels, T = 4.91, *p*_FWE_ = 0.02) (**Figure 4**). The longer the OC use duration, the stronger the deactivation of these areas during navigation.

**Figure 5:**
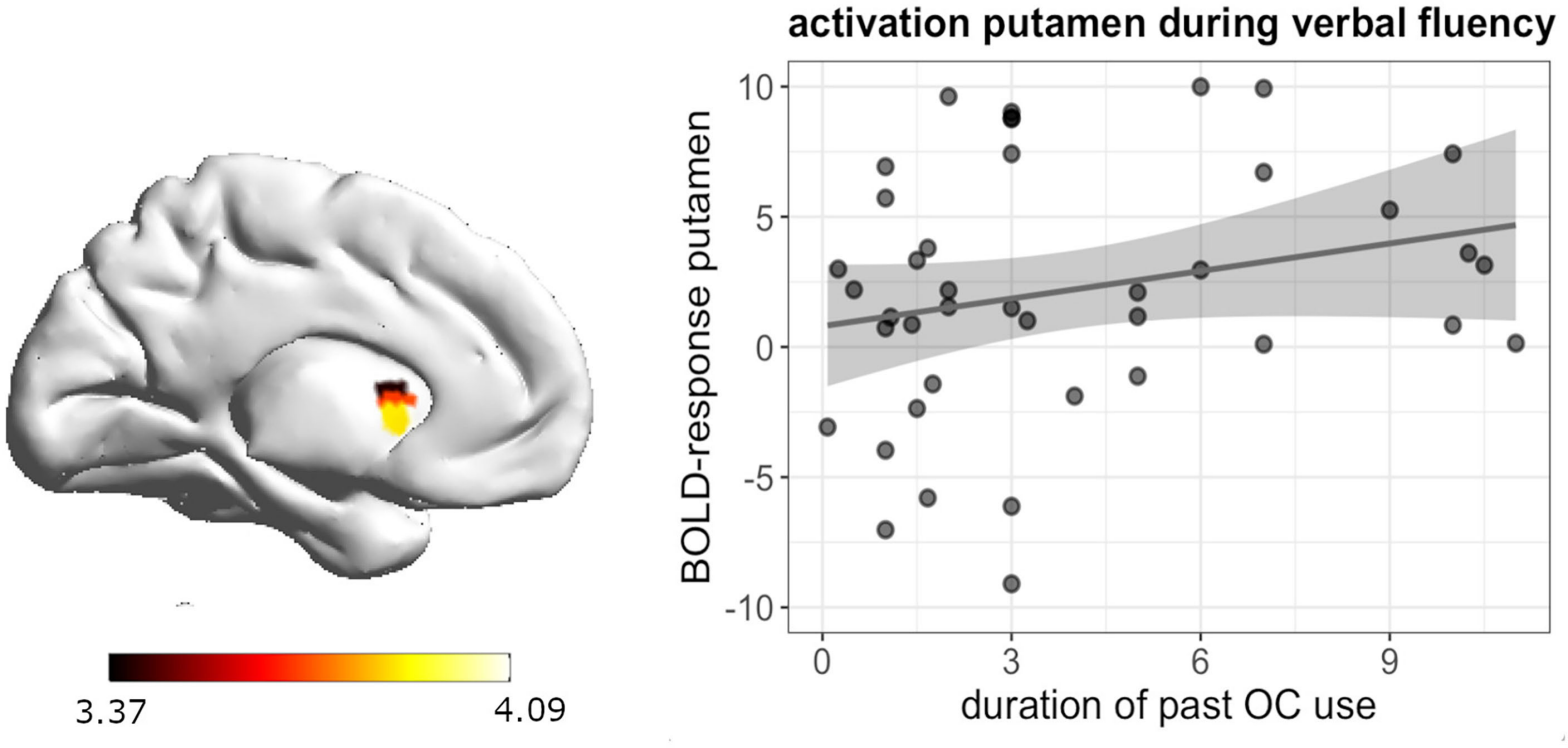
Positive association between duration of OC and left putamen activation during verbal fluency in past OC users.

During verbal fluency, whole brain analyses revealed a significant positive association between the duration of previous androgenic and anti-androgenic OC use and activation in the left putamen ([-24, 8, -8], 48 voxels, T = 4.09, *p*_FWE_ = 0.047) (**Figure 5**). The longer women had previously taken OCs, the stronger was their activation in the left putamen during verbal fluency irrespective of androgenicity.

### Inter-hemispheric connectivity: ROI-to-ROI analysis

**Figure 6:**
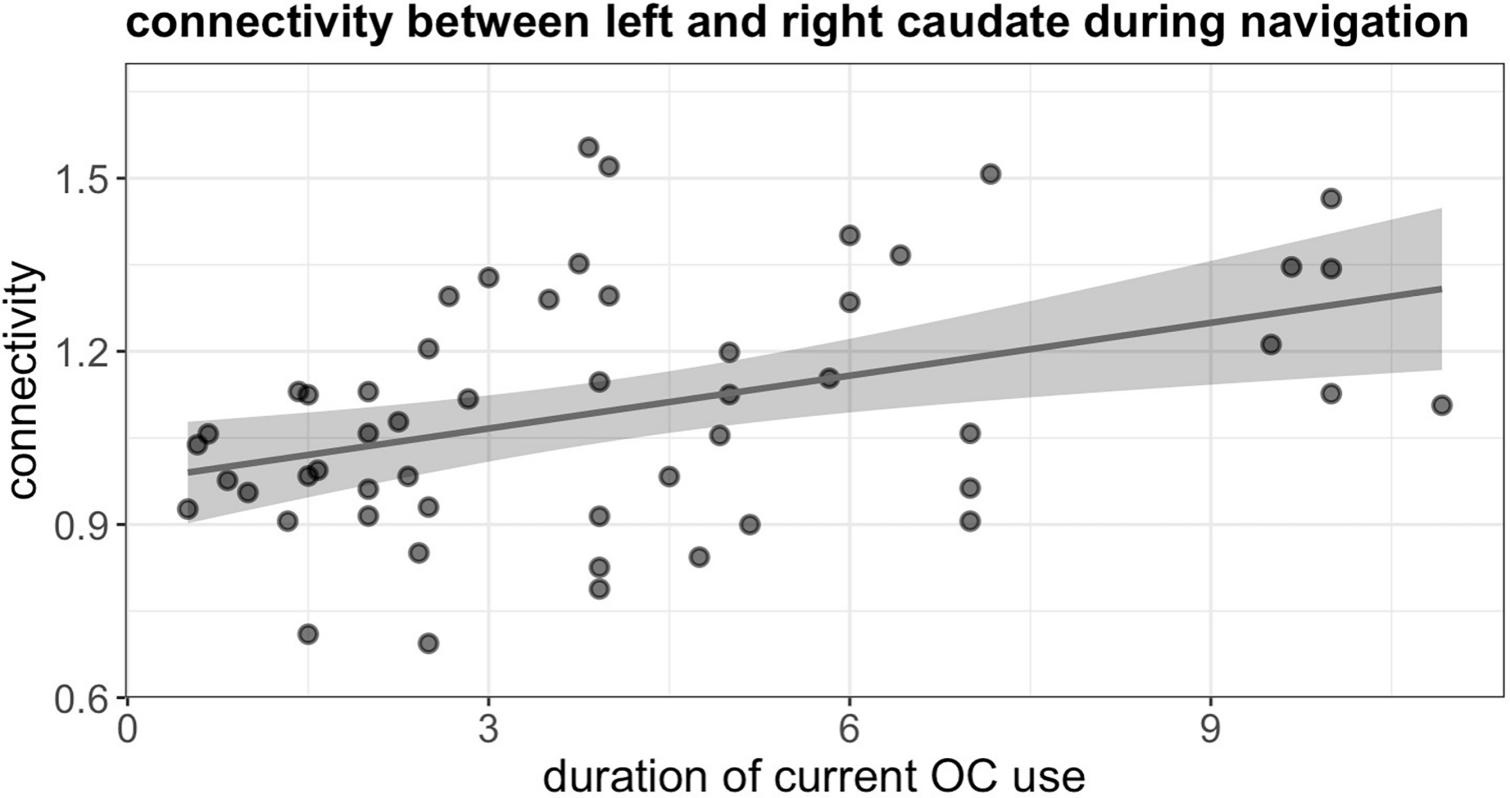
Positive association between duration of current OC use and connectivity between left and right caudate during navigation in current users.

During navigation, the duration of current OC use was significantly associated with stronger connectivity between left and right caudate, irrespective of androgenicity (*b* = 0.49, SE_b_ = 0.17, *t*_(48)_ = 2.84, *p_uncorr._* = 0.007, *p_FDR_* = 0.026) (**Figure 6**).

We observed no effect of pill duration in current and past users on connectivity between left and right hippocampus, left and right DLPFC, left and right IFG as well as in past users on connectivity between left and right caudate during navigation (all b < 0.32, all t < 1.8, all p > 0.05).

Duration of OC use was also not associated with changes in connectivity between left and right hippocampus, left and right caudate, left and right DLPFC and left and right IFG in current nor past users during verbal fluency (all b < 0.36, all t < 1.12, all p > 0.05).

### Functional connectivity: Seed-to-voxel analysis

Seed to voxel analyses revealed no associations between the duration of pill use and connectivity of the right hippocampus, left and right caudate, left and right DLPFC, as well as left IFG, and irrespective of androgenicity. However, during both tasks, connectivity of the left hippocampus was significantly associated with the duration of OC use, irrespective of androgenicity.

**Figure 7:**
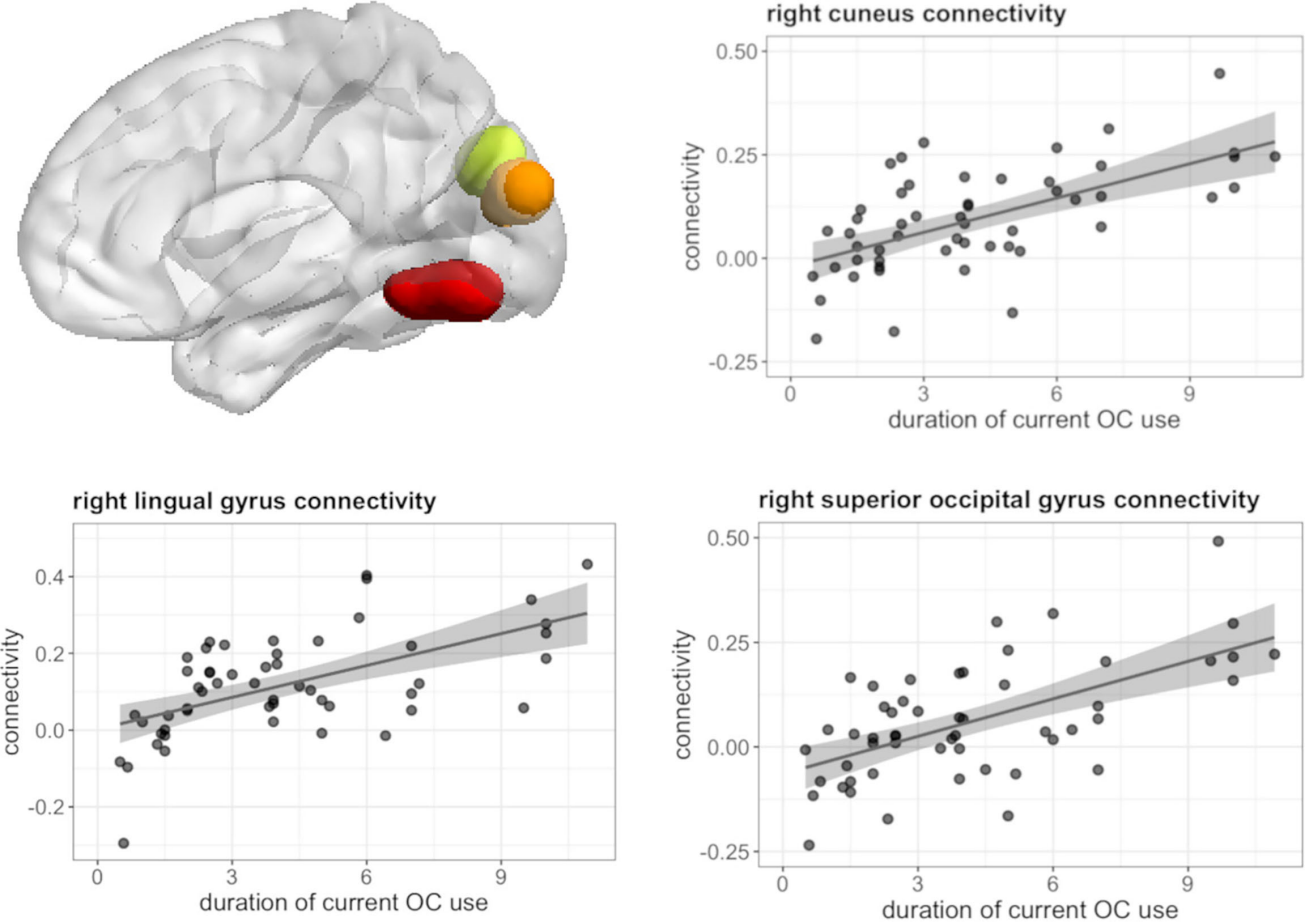
Positive association between duration of OC use and connectivity between left hippocampus and right cuneus, lingual gyrus, superior occipital gyrus in current users during navigation.

**Figure 8:**
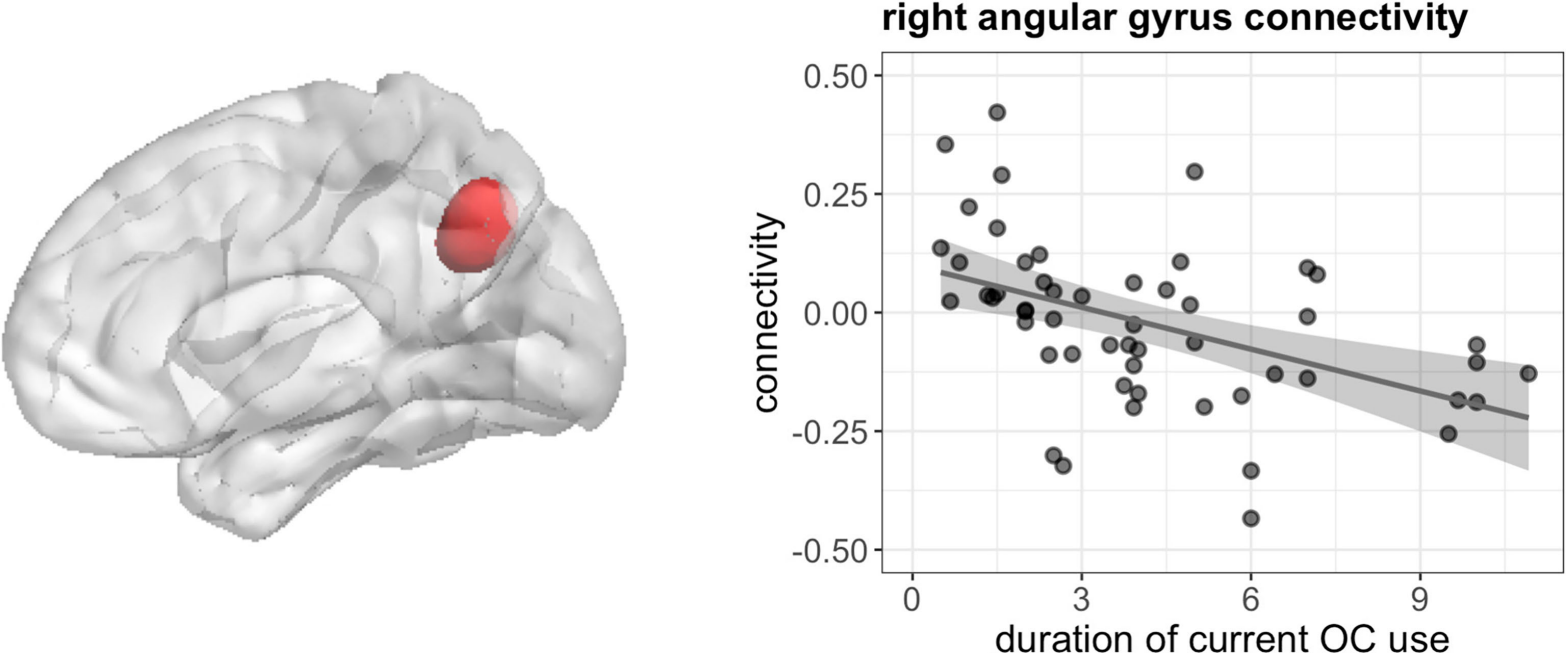
Negative association between duration of OC use and connectivity between left hippocampus and right angular gyrus in current users during navigation.

During navigation, longer duration of current OC use irrespective of androgenicity was associated with stronger connectivity between left hippocampus and right cuneus ([12, -85, 22], 44 voxels, T = 5.80, *p*_FWE_ < 0.001), right lingual gyrus ([27, -58, -11], 85 voxels, T = 5.71, *p*_FWE_ < 0.001) and right superior occipital gyrus ([24, -79, 31], 14 voxels, T = 4.68, *p*_FWE_ = 0.026) (**Figure 7**), as well as weaker connectivity between left hippocampus and right angular gyrus ([42, -55, 34], 49 voxels, T = 5.64, *p*_FWE_ < 0.001) (**Figure 8**). We observed no associations between the duration of past OC use and left hippocampus connectivity during navigation.

**Figure 9:**
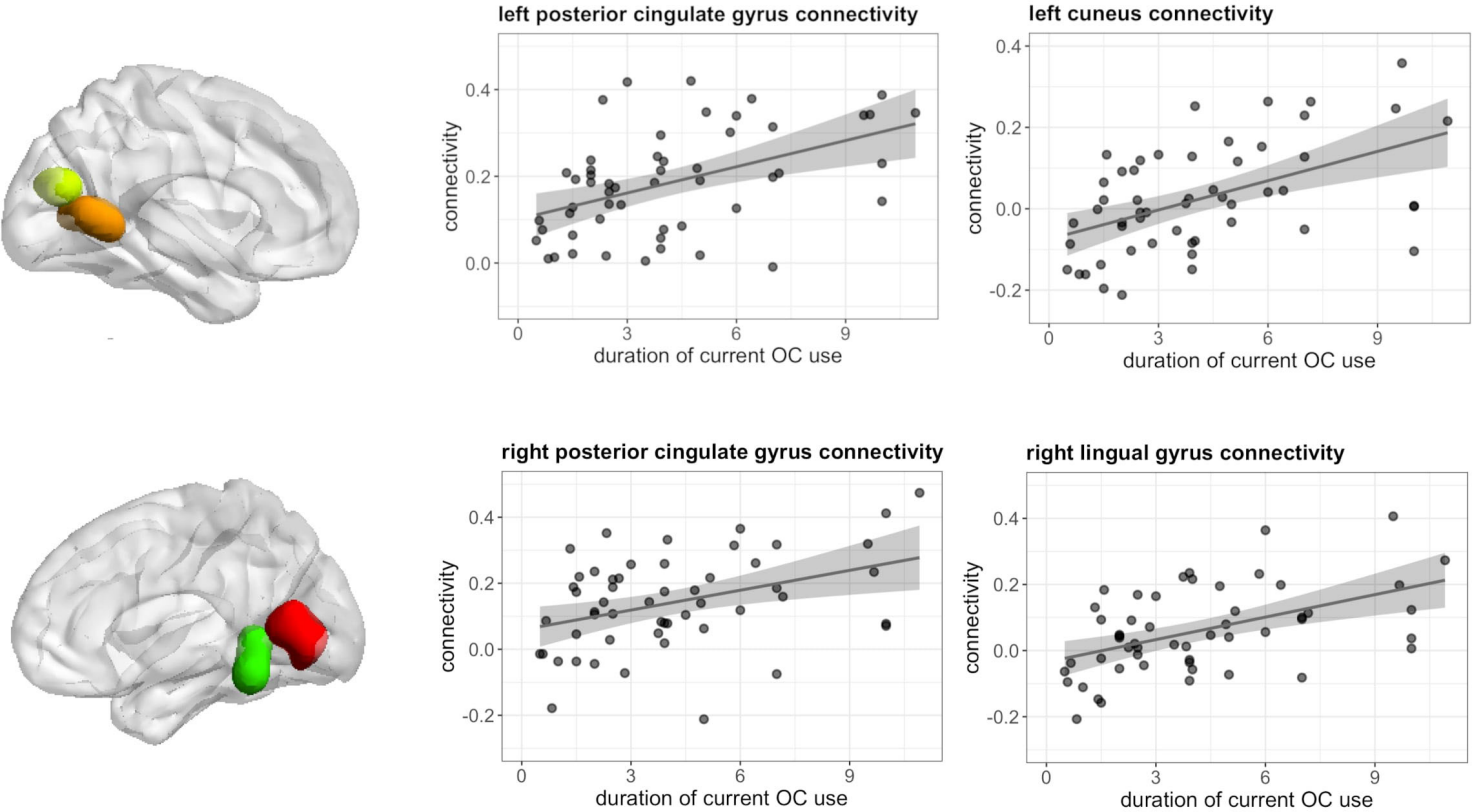
Positive association between duration of OC use and connectivity between left hippocampus and bilateral posterior cingulate gyrus, left cuneus and right lingual gyrus in current users during verbal fluency.

During verbal fluency, longer duration of current OC use, irrespective of androgenicity was associated with stronger connectivity between left hippocampus and bilateral posterior cingulate gyrus (left: [-9, -52, 4], 82 voxels, T = 6.36, *p*_FWE_ < 0.001; right: [18, -46, 1] 19 voxels, T = 4.91, *p*_FWE_ = 0.008), right lingual gyrus ([9, -64, 7], 114 voxels, T = 5.14, *p*_FWE_ < 0.001) and left cuneus ([-9, -73, 19], 22 voxels, T = 4.94, *p*_FWE_ = 0.004) (**Figure 9**).

**Figure 10:**
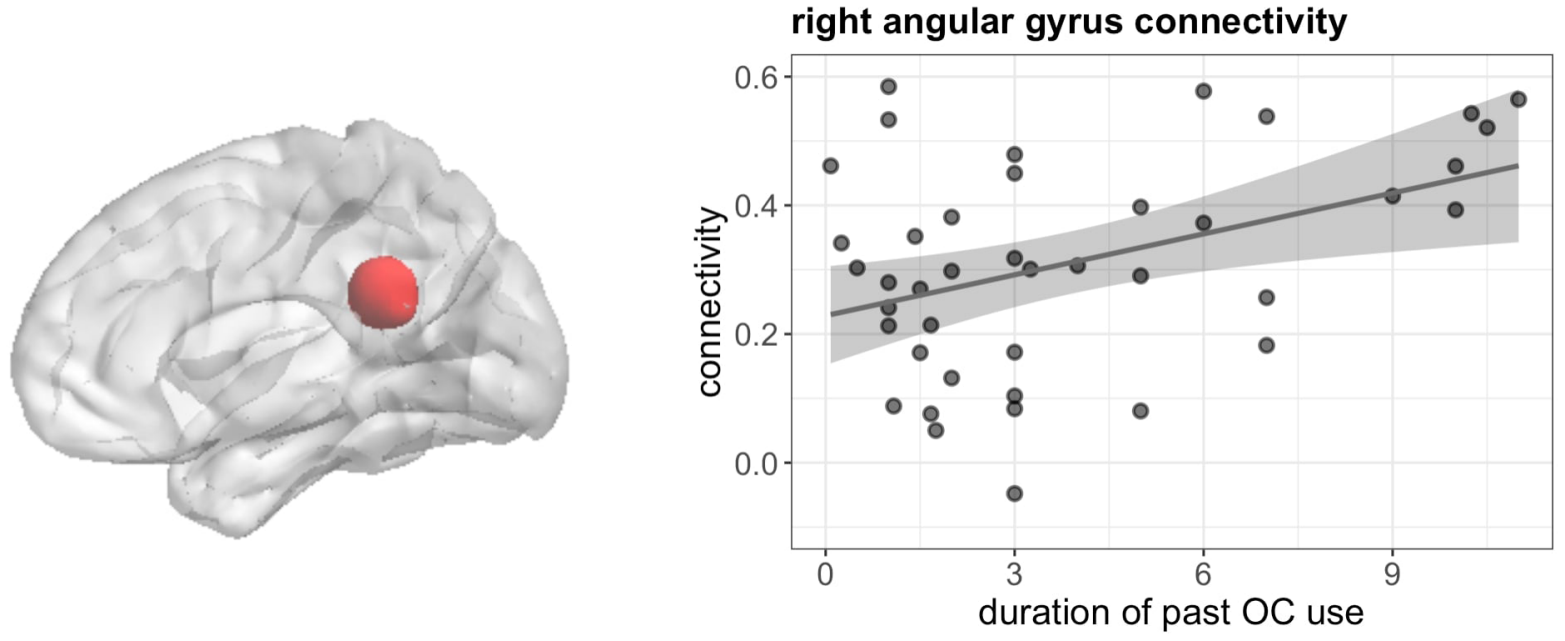
Positive association between duration of OC and connectivity between right IFG and right angular gyrus in past users during verbal fluency.

Furthermore, duration of previous OC use was related to stronger connectivity between right IFG and right angular gyrus during verbal fluency ([51, -43, 28], 50 voxels, T = 6.06, *p*_FWE_ < 0.001) irrespective of androgenicity (**Figure 10**).

## Discussion

The current study explored association between the duration of OC use and cognitive performance, as well as brain activation and connectivity during two different cognitive tasks, that were previously shown to be sensitive to the effects of endogenous sex hormones (7, 40). We further controlled for the androgenicity of the OCs to dissociate effects related to the androgenic activity of progestins from effects related to the estrogenic/progestogenic activity of OCs. Furthermore, we extended the analyses to previous OC users in order to explore the reversibility of OC-dependent effects on the brain. Along these lines, the following commonalities among the results for the navigation and verbal fluency tasks seem noteworthy.

First, several associations to the duration of OC use in the navigation task appear to be modulated by androgenicity with stronger effects in androgenic compared to anti-androgenic OC users, while none of the associations observed for the verbal fluency task were modulated by androgenicity. While the effects of sex hormones to spatial and verbal processing are not entirely understood, several studies hint at an activational role of androgens for spatial processing (69, 70). With regards to verbal memory, results so far point to an important role of estrogens (71–73), while results on the role of androgens during verbal processing remain inconclusive (38, 74–76). In light of these findings, it seems plausible that the androgenicity of progestins affects associations to OC use during spatial processing, but not verbal processing. In particular, deactivation in the caudate and postcentral gyrus appears to be associated to longer androgenic, but not anti-androgenic OC-use. Animal studies hint at different navigation strategies supported by the hippocampus and caudate. While the hippocampus supports “spatial” strategies, the caudate supports stimulus-response learning (77–79). Allocentric landmark-based navigation, as required by the task-instructions in the present study, should per se involve more spatial rather than stimulus-response strategies. However, the current results suggests that the caudate becomes less involved with longer duration of androgenic OC use. The results in the postcentral gyrus are particularly interesting since a recent study on rats found a new spatial navigation system in the somatosensory cortex (80). The authors detected functionally distinct spatial cell types like place, grid and head-direction cells in the primary somatosensory cortex which is located in the postcentral gyrus. The cells showed spatially selective firing patterns which are similar to the ones of the hippocampal place cells. This finding indicates the existence of a spatial map system in rodents’ sensory cortices (80).

Second, in the navigation task, no associations to previous OC use were observed, while in the verbal fluency task, duration of previous OC use affects a variety of brain parameters, including activation of the left putamen, as well as connectivity between right-hemispheric language areas, i.e. the right IFG and the right AG. This points towards stronger organizational effects of OCs on verbal rather than spatial processing. While it is hard to disentangle organizational from activational effects of sex hormones, several results suggest that hormonal effects on spatial processing are of a more activational, while effects of sex hormones on verbal processing are of a more organizational nature. For instance, the onset of sex differences in spatial abilities appears to be roughly around puberty (though social factors may also contribute to this finding), while sex differences in verbal abilities are observed from a very young age on (38). It is however also possible that results in previous OC users were missed due to the heterogeneity of progestins used in this group, while current androgenic OC users were all users of levonorgestrel.

A further difference between the navigation and verbal fluency task are the associations of current OC use to task performance. While duration of OC use was not related to task performance in the navigation task, verbal fluency performance was slightly reduced with longer duration of current OC use. It has to be noted that – similar to previous neuroimaging studies using these tasks (e.g. 7) – behavioral results should be interpreted with caution, since our tasks were optimized for the assessment of brain activation and may not be sensitive enough to reflect changes in behavioral performance. For example, the strong inter-individual variability in navigation performance requires a fixed length of navigation trials, which makes an assessment of navigation speed imprecise. Likewise, the verbal fluency task had to be implemented as a covert version to avoid movement artefacts. Nevertheless, the results may hint that OC effects on the brain during navigation are adaptive and may represent altered processing styles to uphold task performance. This interpretation seems plausible, given that both caudate activation and connectivity as well as hippocampus connectivity were related to the duration of current OC use. As discussed above, these two areas have been implicated in the balancing of different processing styles during navigation. The fact that the duration of OC use is reflected in task performance during verbal fluency may also be in line with the interpretation of more organizational effects during verbal fluency.

Surprisingly, previous studies indicate protective effects of estrogens on verbal and memory performance (71, 73), while verbal fluency performance was impaired with longer OC use in the current study. However, many studies proposing a protective effect of synthetic estrogens have been conducted in post/menopausal women and the suppression of endogenous estrogen fluctuation by the OC use could be responsible for our findings. It is still difficult to disentangle the effects of the lack of endogenous hormones from effects of synthetic OC hormones. Apart from that, an alternative explanation could be the progestagenic actions of OCs since a study on premenopausal women found an association between decreased verbal memory performance and high dose of progesterone intake (81). This placebo-controlled study on the effects of oral progesterone on verbal memory found that the highest dose of 1200mg was associated with decreased information processing and verbal memory performance (81). However, there are contradicting results on the effect of progesterone on verbal memory (82). The idea of an increased effort with the retrieval of verbal information from long-term memory with longer OC use may also be supported by the increased connectivity between the left hippocampus and PCC, which is a major integrating hub and default mode area associated with cognitive effort and task demands (83, 84). Another factor influencing the cognitive effect of OC use might be the vulnerability to depressive episodes (85) since depressive symptoms are associated with compromised cognition (86). Although recent studies have related previous OC use to the development of mood disorders (87, 88), it is still unclear if OC use causes an increased vulnerability to depression (89).

Finally, the results we observed in the hippocampus deserve some attention, given that this very plastic area has been suggested as most sensitive to the effects of sex hormones by both animal and human studies (90, 91). While previous studies very consistently suggest stronger hippocampal activation during high estrogen phases irrespective of the cognitive task involved (5, 7), no effects of OC use duration on hippocampal activation were observed. This is likely due to OCs representing a combination of estrogenic and progestagenic treatment, as observed, e.g., during the luteal cycle phase, while previous results on hippocampal activation were observed during the pre-ovulatory phase, characterized by high estradiol, but low progesterone. Pre-clinical studies provide numerous examples of opposite effects of estrogens and gestagens on various neurotransmitter systems, including the glutamatergic and GABA-ergic systems. Accordingly, estrogens are ascribed excitatory qualities, while progestogens appear to oppose the excitatory effects of estrogens and display inhibitory effects. It is likely that the progestagenic actions of progestins contained in OCs counteract the excitatory effects of estrogens on the hippocampus.

Even though duration of OC use was not significantly associated with activation in the hippocampus, left hippocampal connectivity was substantially modulated by OC duration in both tasks. Irrespective of the task, a stronger connectivity between the hippocampus and various occipital areas was observed. It can be very carefully speculated that this may be related to the transfer of visual information to long-term memory. The reduced connectivity between the left hippocampus and right angular gyrus during navigation fits to the pattern pointing to a shift in strategy or processing style during navigation. Previous studies using this task suggested a stronger verbal labelling of landmark information in women compared to men, which was also reflected in the stronger recruitment of language areas by the hippocampus (40). It is possible that the reduced connectivity between the hippocampus and angular gyrus reflects a reduced recruitment of language areas by the hippocampus.

The main limitation of this study is its correlational nature. While it goes beyond traditional cross-sectional designs and we controlled by confounds like age, education or IQ, we cannot make inferences about the causality of these effects but rather view our results as hypothesis-generating for future prospective studies. Apart from the restrictions regarding behavioral measures and various progestins used among previous contraceptives, another interesting factor to consider in future studies is the age at first contraceptive intake, since developing brains may be more sensitive to synthetic steroids (e.g., 35).

In summary, the current study reveals adaptive brain associations of current OC treatment duration on spatial processing, which depend on the androgenicity of the progestin and are suggestive of a potential shift in processing style with long-term contraceptive use, particularly in the case of androgenic OCs. Furthermore, we observed brain associations of both current and previous OC treatment duration on verbal processing, which are accompanied by a small, but consistent drop in verbal fluency performance. Compared to previous findings from studies on endogenous sex hormones, the results suggest a key role of the progestogenic component of OCs in both tasks.

## Data availability statement

Data and scripts for ROI-analyses are openly available at https://osf.io/q7wth/ and http://webapps.ccns.sbg.ac.at/OpenData/. MR-images for whole-brain analyses are available from thecorresponding author upon reasonable request.

## Ethics statement

The studies involving human participants were reviewed and approved by University of Salzburg’s ethics committee. The participants provided their written informed consent to participate in this study.

## Author contributions

BP designed and made the concept of the study. IN and EH-L were responsible for data acquisition. Analysis of the data was performed by BP, EH-L, and IN. IN drafted the manuscript, which was revised and approved by BP. All authors agree to be accountable for all aspects of the work in ensuring that questions related to the accuracy and integrity of any part of the work are appropriately investigated and resolved. All authors contributed to the article and approved the submitted version.

## Funding

This study was funded by FWF project P337276 and the European Research Council (ERC) Starting grant 850953. IN was supported by the PhD Programme “Imaging the Mind” funded by the Austrian Science Fund (FWF; W 1233-B).

## Acknowledgments

The authors thank all participants and students for their time and willingness to contribute to this study.

## Conflict of interest

The authors declare that the research was conducted in the absence of any commercial or financial relationships that could be construed as a potential conflict of interest.

## Publisher’s note

All claims expressed in this article are solely those of the authors and do not necessarily represent those of their affiliated organizations, or those of the publisher, the editors and the reviewers. Any product that may be evaluated in this article, or claim that may be made by its manufacturer, is not guaranteed or endorsed by the publisher.
