# Supplementary material for: Duration of oral contraceptive use relates to cognitive performance and brain activation in current and past users
Source: Front Endocrinol (Lausanne). 2022 Sep 20;13:885617. doi: 10.3389/fendo.2022.885617 (PMC9530450; doi:10.3389/fendo.2022.885617)
Supplement: Supplementary file 1 [file Table_1.docx]

**Duration of oral contraceptive use relates to cognitive performance and**

**brain activation in current and past users**

Isabel Asar Noachtar^1*^, Esmeralda Hidalgo-Lopez^1^ and Belinda Pletzer^1*^

^1^Department of Psychology and Centre for Cognitive Neuroscience, University of Salzburg, Salzburg, Austria

*corresponding authors:
Isabel Noachtar, isabel.noachtar@gmail.com

Belinda Pletzer, belinda.pletzer@plus.ac.at

**Supplementary information**

**Supplementary Table 1: Whole brain activation during navigation & verbal fluency for the whole sample (N = 94)**

| **Brain region** | **Side** | **MNI-coordinates (mm)** | | | **#voxels** | ***T*** | ***Cluster level*** |
| --- | --- | --- | --- | --- | --- | --- | --- |
|  |  | **X** | **Y** | **Z** |  |  | ***p_FWE_*** |
| **Navigation** |  |  |  |  |  |  |  |
| Superior Frontal Gyrus | L | -24 | -4 | 55 | 683 | 19.04 | <0.001 |
| Superior Frontal Gyrus | R | 24 | -4 | 52 | 306 | 18.41 | <0.001 |
| Supplementary Motor Cortex | L | -6 | -8 | 49 | 288 | 11.06 | <0.001 |
| Superior Parietal Lobe | L | -18 | -58 | 61 | 3195 | 20.46 | <0.001 |
| Anterior Insula | L | -30 | 20 | 1 | 179 | 14.61 | <0.001 |
| Anterior Insula | R | 30 | 23 | -2 | 208 | 15.65 | <0.001 |
| **Verbal fluency** |  |  |  |  |  |  |  |
| Supplementary Motor Cortex | L | -3 | 11 | 49 | 187120 | 36.26 | <0.001 |
| Supramarginal Gyrus | R | 48 | -34 | 46 | 68 | 8.90 | 0.022 |
